# Web-Based Peer Support Interventions for Adults Living With Chronic Conditions: Scoping Review

**DOI:** 10.2196/14321

**Published:** 2021-05-25

**Authors:** Saima N Hossain, Susan B Jaglal, John Shepherd, Laure Perrier, Jennifer R Tomasone, Shane N Sweet, Dorothy Luong, Sonya Allin, Michelle L A Nelson, Sara J T Guilcher, Sarah E P Munce

**Affiliations:** 1 Toronto Rehabilitation Insititute University Health Network Toronto, ON Canada; 2 Rehabilitation Sciences Institute University of Toronto Toronto, ON Canada; 3 Department of Physical Therapy University of Toronto Toronto, ON Canada; 4 Institute of Health Policy, Management, and Evaluation University of Toronto Toronto, ON Canada; 5 University of Toronto Libraries University of Toronto Toronto, ON Canada; 6 School of Kinesiology and Health Studies Queen's University Kingston, ON Canada; 7 Collaboratory for Research and Innovation Lunenfeld-Tanenbaum Research Institute Toronto, ON Canada; 8 Leslie Dan Faculty of Pharmacy University of Toronto Toronto, ON Canada; 9 Toronto Rehabilitation Institute - Rumsey Centre University Health Network Toronto, ON Canada

**Keywords:** online, peer support, self-management, chronic conditions, scoping review

## Abstract

**Background:**

Globally, 1 in 3 adults live with multiple chronic conditions. Thus, effective interventions are needed to prevent and manage these chronic conditions and to reduce the associated health care costs. Teaching effective self-management practices to people with chronic diseases is one strategy to address the burden of chronic conditions. With the increasing availability of and access to the internet, the implementation of web-based peer support programs has become increasingly common.

**Objective:**

The purpose of this scoping review is to synthesize existing literature and key characteristics of web-based peer support programs for persons with chronic conditions.

**Methods:**

This scoping review follows the PRISMA (Preferred Reporting Items for Systematic Reviews and Meta-Analyses) extension for scoping reviews guidelines. Studies were identified by searching MEDLINE, CINAHL, Embase, PsycINFO, and the Physiotherapy Evidence Database. Chronic diseases identified by the Public Health Agency of Canada were included. Our review was limited to peer support interventions delivered on the web. Peers providing support had to have the chronic condition that they were providing support for. The information abstracted included the year of publication, country of study, purpose of the study, participant population, key characteristics of the intervention, outcome measures, and results.

**Results:**

After duplicates were removed, 12,641 articles were screened. Data abstraction was completed for 41 articles. There was a lack of participant diversity in the included studies, specifically with respect to the conditions studied. There was a lack of studies with older participants aged ≥70 years. There was inconsistency in how the interventions were described in terms of the duration and frequency of the interventions. Informational, emotional, and appraisal support were implemented in the studied interventions. Few studies used a randomized controlled trial design. A total of 4 of the 6 randomized controlled trials reported positive and significant results, including decreased emotional distress and increased health service navigation, self-efficacy, social participation, and constructive attitudes and approaches. Among the qualitative studies included in this review, there were several positive experiences related to participating in a web-based peer support intervention, including increased compassion and improved attitudes toward the individual’s chronic condition, access to information, and empowerment.

**Conclusions:**

There is limited recent, high-level evidence on web-based peer support interventions. Where evidence exists, significant improvements in social participation, self-efficacy, and health-directed activity were demonstrated. Some studies incorporated a theoretical framework, and all forms of peer support—emotional, informational, and appraisal support—were identified in the studies included in this review. We recommend further research on web-based peer support in more diverse patient groups (eg, for older adults and chronic conditions outside of cancer, cardiovascular disease, and HIV or AIDS). Key gaps in the area of web-based peer support will serve to inform the development and implementation of future programs.

## Introduction

### Background

In Canada, 1 in 5 adults live with cardiovascular disease, cancer, diabetes, or chronic respiratory disease [1]. These chronic conditions account for 65% (153,000) of deaths in Canada each year [2] and are the leading causes of death globally [1]. These chronic conditions account for 42% of direct health care costs in Canada or Can $39 billion (US $32 billion) per year [2]. The total economic burden is a combination of medical costs (Can $38.9 billion; US $31.9 billion) and indirect productivity losses (Can $54.4 billion; US $44.6 billion) [2]. Globally, 1 in 3 adults live with multiple chronic conditions [3], and among Americans aged ≥65 years, approximately 3 in 4 adults have multiple chronic conditions [4]. Thus, effective interventions are needed to prevent and manage these chronic conditions and to reduce the associated health care costs.

Teaching effective self-management practices to people with chronic diseases is one strategy to address the burden of chronic conditions [4]. For example, in the United States, the Affordable Care Act encourages chronic disease self-management practices [5]. The Affordable Care Act offers reimbursement opportunities for providers of chronic disease management services and provides government support for the development of programs aimed at self-management [5]. In the context of chronic conditions, self-management refers to a patient’s ability to manage various physical and psychosocial ailments and lifestyle changes [6,7]. Previous research has indicated that peers can support chronic disease self-management [8] in a cost-effective manner [8-10]. For example, an economic evaluation conducted by Graffy et al [11] found lower total health care costs due to decreased hospitalization expenses among individuals with diabetes who had received peer support (group or one-to-one delivery) compared with those among control groups.

In the context of chronic disease management, peer support refers to providing assistance to other individuals with similar conditions [8,11]. Programs with an associated peer support component have 3 commonalities: support for emotional, informational, and appraisal needs [12]. Emotional support includes caring, empathy, and encouragement of the individual. Informational support refers to providing advice, suggestions, and alternative actions. Appraisal support involves affirmation, constructive feedback, and the provision of information useful for self-evaluation [13]. Peer support programs can be delivered using a wide variety of modalities, including face-to-face, telephone, or internet. With the increasing availability and access to the internet (eg, over 32 million people in Canada [14] and 55.1% of the world’s population [15]), the implementation of web-based peer support programs, in particular, has become increasingly common and relevant [14-16].

### Objective

With the increasing implementation of web-based peer support interventions, there is a need to examine the characteristics of these interventions and determine the gaps in this emerging literature. The purpose of this scoping review is to synthesize the existing literature and key characteristics (eg, duration; frequency; delivery setting; type of intervention; type of support provided, including emotional, informational, and appraisal; and underlying theories for the intervention, behavior change techniques, or mechanisms) of web-based peer support programs for persons with chronic conditions.

## Methods

### Overview

The methodology for this scoping review has been previously published [17], but it is briefly described below. This scoping review follows the PRISMA (Preferred Reporting Items for Systematic Reviews and Meta-Analyses) extension for scoping reviews guidelines [18].

### Search Strategy and Information Sources

A comprehensive literature search was conducted by an experienced librarian (LP) with input from the investigators. Literature search strategies were developed using medical subject headings and text words related to chronic conditions and peer support interventions. The MEDLINE search has been previously published in our protocol paper [17]. The search was initially run on May 6 and 8, 2017, and rerun on June 6, 2018. The following databases were searched: MEDLINE (OVID), MEDLINE In-Process & Other Non-Indexed Citations (OVID), MEDLINE Epub Ahead of Print (OVID), Embase (OVID), CINAHL (EBSCOhost), Physiotherapy Evidence Database, and PsycINFO (OVID). A validated search filter for identifying age-specific studies that specifically identified citations for adults was added to MEDLINE, Embase, and CINAHL. Duplicates were removed by using EndNote’s duplicate identification feature and by reviewing records manually. Searches were limited to studies conducted from 2012 to 2018 and English language studies. Studies were included from this 6-year window to increase the relevance to the current health care context. Due to time and resource constraints, we were unable to extend the search beyond this 6-year window. In addition, for feasibility considerations, no hand searching was performed.

### Eligibility Criteria

Chronic diseases identified by the Public Health Agency of Canada (PHAC), including cancer, heart disease (cardiovascular disease), hypertension, stroke, chronic respiratory diseases (asthma, chronic obstructive pulmonary disease, and sleep apnea), diabetes, inflammatory bowel diseases (Crohn disease and ulcerative colitis), multiple sclerosis, neurological conditions (eg, Alzheimer disease and other dementias), cerebral palsy, epilepsy, multiple sclerosis, Parkinson disease or parkinsonism, traumatic brain injury, traumatic spinal cord injury, arthritis, and osteoporosis, were included [19]. This list of chronic conditions is consistent with other global definitions of chronic conditions (eg, the World Health Organization) [20]. This review included studies involving individuals with chronic conditions, including comorbid mental illness. Studies must have reported on adults (age≥18 years) with one of the previously listed PHAC chronic conditions or HIV or AIDS. Although mental illness is included in the PHAC list of chronic diseases, it was excluded for the purposes of this review because peer support interventions for this specific group may have unique features (eg, coping with stigma, including self-blaming, guilt, and shame) that may not be generalizable to other patient populations with chronic disease [21,22]. Similarly, although not included in the PHAC list, due to the high volume of web-based peer support interventions reported on individuals with HIV or AIDS, it was included in this review’s list of chronic diseases [23]. In addition, including HIV or AIDS in this list of inclusion criteria was further rationalized by a similar review conducted by Lauckner et al [12], who examined peer support for people with chronic conditions in rural areas.

Our review was limited to peer support interventions delivered on the web. Studies were included if a web-based peer component was part of their intervention. Support must be provided by a peer who has the same chronic condition. Examples of web-based peer interventions include video-based discussions using formats such as Skype, social media peer interactions, and text messages from peers. Peer-led interventions that used a web-based modality in combination with another modality, such as telephone or face-to-face interventions, were included. Interventions describing professional-led groups involving community health workers who are not peers (eg, health care professionals), e-counseling service interventions, studies reporting on outcomes of usability testing but not the outcomes of the participants, support group interventions, and telephone-based peer support interventions were excluded. In addition, studies were excluded if they described the benefits of using the internet generally but did not describe an intervention and the reported outcomes of that intervention. If the study described an intervention that had a combination of peer- and professional-led support, it was excluded.

To further describe the types of articles that were included and excluded in this review, we provide an example of 1 study that was included and 2 that were excluded.

The study *Development of Trust in an Online Breast Cancer Forum: A Qualitative Study* by Lovatt et al [24] was included in this review. This study explored the breast cancer care forum by collecting discussion threads and analyzing them. In this case, the web-based forum was the modality for delivering peer support. The study *The Emerging Diabetes Online Community* by Hilliard et al [25] was excluded from our study. Although the study reported on multiple web-based platforms (eg, forums, blogs, video or podcasts, and social media websites used by individuals living with diabetes), the study did not report on the outcomes or experiences of a specific web-based peer support intervention. Finally, the study *Online support for individuals with spinal cord injuries: An ethnographic investigation* by O’Riley et al [26] was excluded. This study involved interviews to explore how individuals with a spinal cord injury could benefit or might use the internet for support. However, no specific peer support intervention had been implemented. Finally, all study designs (eg, observational studies, randomized controlled trials, and qualitative studies) were included.

### Study Selection

The studies were screened using a 2-step process. First, the titles and abstracts were screened in duplicate by independent reviewers, followed by full-text screening, which was conducted in duplicate. Both level 1 and level 2 screening followed the same screening form. DistillerSR reference manager was used by independent reviewers to keep track of the decisions. Discrepancies were resolved by discussion between reviewers and, if necessary, the senior author (SEPM).

### Data Abstraction

Data abstraction forms developed by the research team were used. The information abstracted included year of publication, country of study, purpose of the study, participant population (eg, chronic condition, age, sex, gender, and education), key characteristics of the intervention (eg, duration; frequency; delivery setting; type of intervention; type of support provided, including emotional, informational, and appraisal; underlying theories for the intervention, behavior change techniques, or working mechanisms; and context), outcome measures, and results. Results including *P* values were collected for the quantitative studies; themes and subthemes were abstracted for the qualitative studies. For qualitative studies, similar themes across studies were clustered together by the lead author (SNH) in consultation with the senior author. Data abstraction was conducted independently in duplicate.

## Results

### Overview

**Figure 1:**
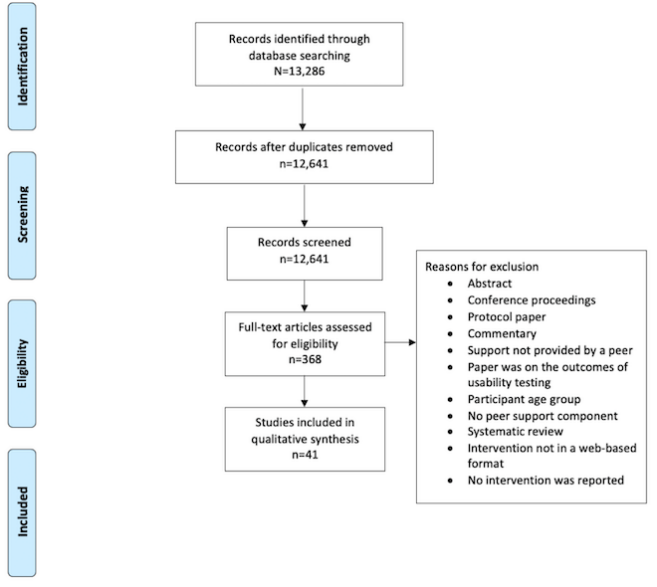
PRISMA (Preferred Reporting Items for Systematic Reviews and Meta-Analyses) flowchart.

The literature searches yielded 13,286 articles. After duplicates were removed, 12,641 articles were screened. After level 1 screening, 368 articles were included in the full-text screening. Of these 368 items, 5 oral presentations and 37 abstracts from conferences were excluded, as it was not possible to obtain full-text articles. A total of 9 protocol papers were excluded because there were no data on the results of the reported interventions. After level 2 screening, data abstraction was completed for 41 articles. The reasons for article exclusion varied but were primarily related to not having a peer support component implemented in the studied intervention. Further rationale as to why articles were excluded are described above within the *Eligibility Criteria* section. The PRISMA flowchart is shown in Figure 1.

### Study Characteristics

A summary of the included studies with information on patient characteristics, peer support intervention characteristics, outcome measures, and impact is included in Multimedia Appendix 1 [24,27-65]. A total of 18 studies were conducted in the United States, 5 in Australia, 5 in the United Kingdom, and 4 in Canada. The remaining 9 studies were conducted in Finland, Sweden, Germany, China, Italy, South Africa, and Sweden.

Around half of the studies included in this review were qualitative (20/41, 49%). Specifically, 14 of these studies analyzed content posted on web-based discussion forums, whereas the other 6 were qualitative studies that reported on the experiences of the patients participating in a web-based peer support program. The quantitative studies included in the review were randomized controlled trials (6/41, 14%) and cross-sectional studies using a survey design (8/41, 19%). The remaining studies were mixed methods studies (4/41, 10%), nonrandomized controlled trials in which matched controls served as the comparison group, quasi-experimental studies (1/41, 2%), and integrative reviews (1/41, 2%).

There were various limitations to the included studies. Among the randomized controlled trials, there were small sample sizes (sample sizes ranged from 30 to 227) [27-32], weak validity and reliability of the measures included [30], and the type of control group used in the trial (eg, control condition involved usual treatment) [31]. In terms of the limitations of the qualitative studies, some examples included selection bias [33], limited transferability of the study findings [34], and the potential for information to be removed by a moderator [35].

### Patient Characteristics

The studies included participants with cancer (15/41, 36%), diabetes (9/41, 21%), and HIV (7/41, 17%). The remaining 10 studies included participants with arthritis (2/41, 4%), atrial fibrillation (1/41, 2%), chronic pain (1/41, 2%), inflammatory bowel disease (1/41, 2%), multiple sclerosis (2/41, 4%), peripartum cardiomyopathy (1/41, 2%), and stroke (2/41, 4%). The individuals included in the studies were aged between 19 and 70 years. This broad range of age groups made it difficult to summarize the studies based on specific age groups. In the majority of the studies, the ratio of male to female participants varied, except in studies on chronic conditions that are of higher prevalence in a specific sex (eg, breast cancer and prostate cancer) [24,33,36-39,66]. Specifically, of the 3 studies that reported on breast cancer, 2 reported that all participants were female.

### Key Characteristics of the Peer Support Interventions

This section outlines the following key characteristics of the studies on web-based peer support interventions included in our review: duration; frequency; delivery setting; type of intervention; type of support provided, including emotional, informational, and appraisal; and underlying theories for the intervention, behavior change techniques, or mechanisms.

#### Duration and Frequency

For the included studies, the duration and frequency of the interventions varied. Of the 41 included studies, 15 (36%) had interventions lasting for 2 weeks to 16 weeks [27-31,33,40-47,66], whereas some interventions (2/41, 4%) lasted for 1-2 years [48,49]. The frequency of peer interaction ranged from weekly interactions to monthly updates [27,29-31,41,43,46,48].

#### Delivery Setting

A total of 15 of the 41 studies described a web-based discussion board as the means of delivering the intervention [24,32-35,39,40,43,45,50-54,66]. Moreover, 9 of the 41 studies used an existing social network site such as Facebook, Twitter, or Myspace [29,36,37,44,47,48,55-57]. In addition, 6 of the 41 studies described a unique web-based platform that consisted of different components such as information modules, live chats, and web-based discussion boards to create a community of participants involved in web-based discussions [30,31,38,49,58,59]. Furthermore, 5 of the 41 studies used a combination of delivery mechanisms, including Skype, social networking sites, forums, telephone, and face-to-face [42,60-63]. One of the 41 studies used Skype video conferencing [41], whereas another study used another web-based video conferencing software [27]. A total of 2 of the 41 studies used a web-based chatroom interface [64,65], and an additional 2 studies used text messaging as a means of peer support (Multimedia Appendix 2) [28,46].

#### Types of Interventions

Of the included studies, 21 of the 41 studies reported on using a group-type intervention [27,29-33,35,40,43-45,47-49,51, 53,55,57-59,64]. A total of 5 of the 41 included studies had web-based peer support delivered through a one-on-one format [24,28,39,41,46]. In the remaining studies (15/41, 37%), it was unclear whether the type of support was delivered through a group or one-on-one format.

#### Type of Support

No studies have reported on interventions that included only one type of support. Instead, the interventions provided a mix of emotional, informational, and appraisal support. A total of 29 studies failed to define a theoretical framework underpinning the intervention.

#### Underlying Theories

In total, 12 studies included the following underlying theories, models, or approaches: social learning theory [50,58,67], social comparison theory [37,68], social support theory by La Coursiere [42,69], self-management theory by Bandura [28,70,71], Information-Motivation-Behavioral Skills model [46,72], self-efficacy theory [30,70], person-centered care approach [32,73], stress process model [31,74], the concept of human bonding and social support as defined by Namkoong et al [34,75], and the conceptual framework outlined by Dennis [76] (emotional, informational, and appraisal support).

### Outcomes Measures and Impact

Among the randomized controlled trials (6/41, 15%), the outcomes used were participant openness, trust, motivation, knowledge, self-efficacy, self-care behavior levels, social relationships, emotional distress, depression, mastery, self-esteem, social support, and general well-being [27,28,30-32]. The measures included were the Working Alliance Inventory [27], California Psychotherapy Alliance Scale [27], Patient-Reported Outcome Quality of Life subscales of body change [30], Positive Outlook Self-Efficacy Scale [30], Health Education Impact Questionnaire [30], and Well-being Questionnaire [32]. The randomized controlled trials included in this review reported on the following conditions: diabetes (2/6, 33%) [28,32], HIV (2/6, 33%) [29,30], cancer (1/6, 17%) [27], and stroke (1/6, 17%) [31].

A total of 2 of the 6 randomized controlled trials on diabetes management reported no statistically significant differences between groups for self-efficacy, general well-being, or self-care behaviors (eg, general diet, exercise, and smoking) [28,31]; however, a higher level of disease-specific knowledge was reported in the group of participants receiving web-based peer support [28]. The remaining 4 of the 6 randomized controlled trials reported positive and significant results, including increased feelings of acceptance and respect by others, health service navigation, self-efficacy, social participation, and constructive attitudes and approaches and decreased emotional distress [28,46-48].

**Figure 2:**
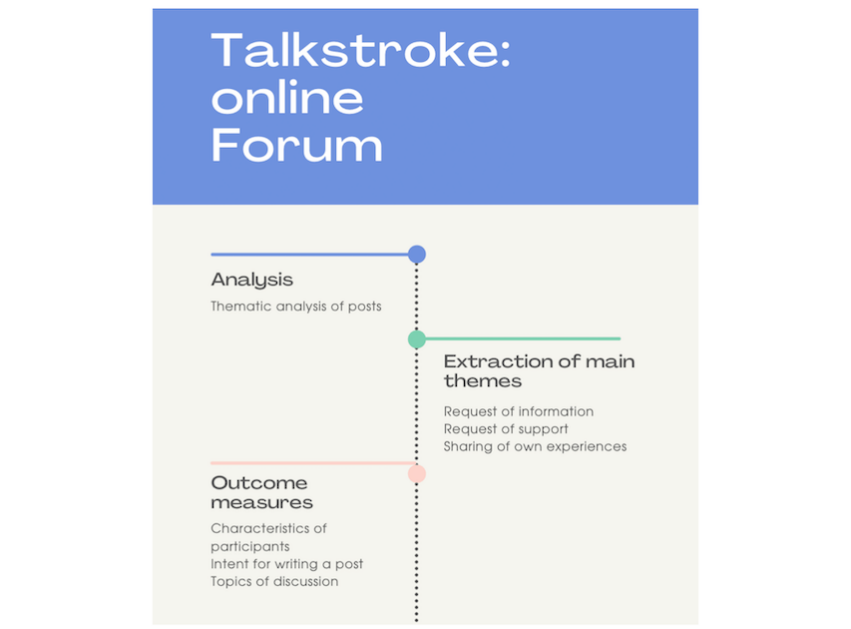
An example of an article included in this study. De Simoni et al [35] reports on web-based stroke forum.

**Figure 3:**
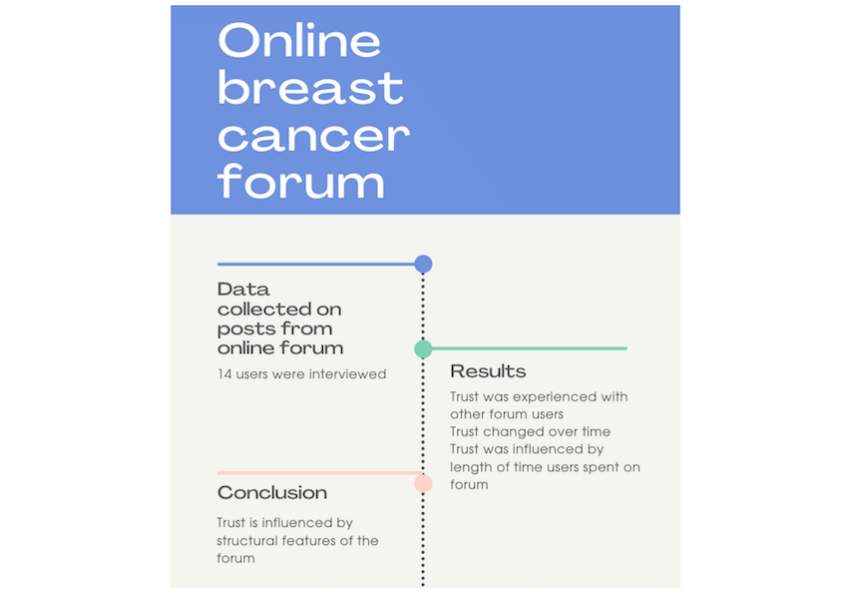
An example of an article included in this study. Lovatt et al [24] reports on the difference between standard of care and web-based support.

In the qualitative studies, some of the positive experiences of participating in an web-based peer support program included increased compassion and improved attitudes toward their condition (ie, people felt that they were not alone in their struggles or that peer support reduced isolation) [38,52], access to information that people could not access through their health care professionals (ie, experiences of people with a similar condition and the gathering of information about a treatment option) [38,52,60], and empowerment (ie, taking an active role in one’s condition) [33]. Among these qualitative studies, several barriers and enablers to obtaining peer support were identified. Some of the barriers to participating in the web-based peer support program included challenges of timing with other life events, a lack of availability or access, the perception of not fitting in with a web-based group, and the need for more condition-specific content [33,38,52]. Enablers to using the program included the use of appropriate language (ie, clear and easy to understand), flexibility or self-pacing, appropriate module length (ie, did not represent a burden to the participant), and the usability of the platform [38,42,48,50]. Furthermore, studies have reported that participants viewed web-based support programs as a unique resource that allows them to be engaged in a program from home anonymously [37,60,65]. To exemplify some of the web-based peer support interventions described in the studies included in this review, we have presented 2 case examples in Figures 2 and 3.

## Discussion

### Principal Findings

#### Overview

This scoping review aims to determine what is known from the existing literature about the key characteristics (eg, duration; frequency; delivery setting; type of intervention; type of support provided, including emotional, informational, and appraisal; and underlying theories for the intervention or behavior change techniques or mechanisms) of web-based peer support interventions for adults with chronic conditions. The main findings of this review were as follows: (1) a lack of participant diversity in the included studies, specifically with respect to the conditions studied (ie, the majority of the studies included were related to cancer, cardiovascular disease, and HIV or AIDS) and the exclusion of older participants (ie, the age range for included studies was 19-70 years); (2) few studies used a randomized controlled trial design; (3) over one-third (15/41, 37%) of the interventions included involved web-based discussion boards, and just over half (21/41, 51%) of the included studies involved group-type peer support; (4) the interventions provided a mix of emotional, informational, and appraisal support, but the majority of studies did not report on an underlying theory or conceptual framework for the intervention; and (5) in terms of outcomes, among the quantitative studies, 4 of the 6 randomized controlled trials demonstrated increased feelings of acceptance and respect by others, health service navigation, self-efficacy, social participation, and constructive attitudes and approaches and decreased emotional distress [28,46-48], whereas for the qualitative studies, participants reported increased compassion and improved attitudes toward their condition [38,52]. To the best of our knowledge, this is the first scoping review to synthesize evidence on web-based peer support interventions across a variety of chronic conditions.

#### Patient Characteristics

Across the 41 included studies, the age range was 19-70 years. Although it is recognized that some conditions are more common among younger individuals (eg, HIV or AIDS), the absence of recent evidence on web-based peer support interventions for individuals aged ≥70 years is noteworthy. For example, in Canada, about 20% of breast cancers are diagnosed in women aged <50 years, whereas almost 30% are diagnosed in women ≥70 years. Individuals ≥70 years are more likely to be socially isolated and lonely [77], and thus, they have the potential to benefit the most from a web-based peer support intervention. For example, in a pre-post pilot study of a peer-to-peer support program engaging older adults to provide companionship to less-able older persons (mean age 69 years) via home visits and phone calls, Geffen et al [78] demonstrated significantly decreased reporting of reduced social interaction and reduced loneliness in addition to increased levels of self-reported well-being, improved emotional and informational support, increased mood scores, and increased levels of physical activity.

The findings of this review suggest that there is a need for more studies on web-based peer support interventions for individuals ≥70 years, in these and other specific disease populations, and for improved methods to target these vulnerable groups. Furthermore, Statistics Canada indicates that rates of internet use vary across age groups within the senior population, with 81% use among older adults aged 65-69 years, compared with 74% use among those aged 70-74 years, 64% use among those aged 75-79 years, and 49% use among those aged ≥80 years [79]. Although it is unclear whether these decreased rates are due to issues related to internet access and/or computer literacy, these potential barriers should be addressed to realize the benefits of web-based peer support (including the benefits outlined in the current review) for older adults with chronic health conditions. In terms of the breadth of chronic diseases included, our results align with a scoping review on peer support for people with chronic conditions in rural areas in terms of identifying studies on a limited range of chronic conditions. Specifically, Lauckner and Hutchinson [12] determined that many studies were related to individuals with diabetes.

#### Duration and Frequency

We determined that there was a lack of consistency in terms of reporting intervention characteristics. Similarly, in a systematic review of peer support interventions for individuals with acquired brain injury, cerebral palsy, and spina bifida, members of our research team concluded that experts from relevant disciplines collaborated to develop the peer support interventions, but they did not specify the methods by which the key components of the interventions such as session duration, frequency, and intervention length were chosen or how these decisions were informed. Given this lack of consistency, it is suggested that future studies reporting on web-based peer support interventions consistently use the better reporting of interventions: a Template for Intervention Description and Replication checklist and guide [80]. This guide includes the following items: brief name, why, what, who provided, how, where, when and how much (ie, duration and frequency), tailoring, modifications, and how well. The application of this checklist could promote the replicability of the intervention and an understanding of the program components that are associated with improved outcomes. At the same time, it is important to recognize that the need to better report the intervention duration and frequency does not apply equally to all web-based peer support contexts. Finally, the number of trials included in this review was too small to draw any associations between the frequency of the peer interactions and the duration of the programs and associated outcomes. However, future trials in this area should examine these associations (ie, dose response).

#### Delivery Setting and Types of Intervention

Other important aspects of this review were the delivery settings and the types of interventions. In terms of the randomized controlled trials, as previously mentioned, the number included was too small to draw any associations between the delivery setting and the type of interventions and outcomes. Among the studies involving nonexperimental designs, 37% (15/41) used web-based discussion boards with a group type of intervention. Group peer-to-peer discussion boards may be particularly valuable, as noted by a qualitative study on the perspectives of individuals with type 1 diabetes using an internet self-management system, as they allow patients to share tips and advice on managing their conditions and provide an opportunity to relate to fellow patients [81]. Similarly, in a qualitative study on one-to-one versus group-based peer support for breastfeeding, Hoddinott et al [82] determined that group-based peer support was more popular, as it normalized breastfeeding in a social environment, which in turn improved participants’ sense of well-being. Participants also indicated that the group format in particular assisted women with decision making [82]. The impact of web-based, one-to-one versus group-based peer support could be the focus of future randomized controlled trials.

#### Types of Support Provided

In our review, we identified all 3 types of support—emotional (eg, communicating a sense of belonging, inclusivity, and reinforcing the presence of others), informational (eg, asking others for guidance and providing detailed explanations), and appraisal (eg, goal setting and action planning that can provide opportunities for constructive feedback)—across the included studies. A review by Lauckner and Hutchinson [12] determined that the majority of programs provided general social support and support related to the development of new skills (eg, appraising health information using a computer; preparing meals; and improving self-management skills, goal setting and problem solving, and general skills to support lifestyle changes). Although they did not identify the specific constructs of emotional, informational, and appraisal support as we did in our review, there appears to be an overlap between the types of support identified in their review and our review, particularly in the areas of informational support (eg, development of new skills) and appraisal support (eg, goal setting and problem solving).

#### Underlying Theories for the Intervention

Only 12 of the 41 studies provided an underlying theory or model or approach, with some of these studies reporting only an underlying approach (ie, person-centered care approach). Previous research suggests that a thorough approach to intervention development, including a clear rationale for the design and development of interventions, is recommended [83,84]. Thus, future peer support interventions should implement an underlying theory or model to inform interventions, which in turn would support the intended outcomes of the intervention.

#### Impact

Lauckner and Hutchinson [12] determined that of the 9 studies that reported on program outcomes, 8 reported positive outcomes, whereas 1 study reported mixed results. Overall program success, participants valuing the social components of the program, improved activity or weight loss, and participants feeling an increased sense of efficacy were the related positive outcomes reported. Similarly, among the trials included in our review, it was demonstrated that web-based peer support programs resulted in improved social participation, self-efficacy, and health-directed activity. Thus, peer support may serve as an important supplement to formal care, as noted by Smith et al [85] in an evaluation of a web-based peer support community intervention. Furthermore, Lauckner and Hutchinson [12] noted that the use of telecommunications with deidentification protocols, such as passwords and pseudonyms, decreased the perceived stigma related to program participation. They also noted that this is particularly important for vulnerable populations. This perspective was noted across many of the included qualitative studies in our review, where participants appreciated the anonymity that a web-based program affords [60,65]. Lauckner and Hutchinson [12] reported that studies that used telecommunications as part of the intervention often provided technical support services to ensure effective program implementation. Similarly, we determined that the availability of technical support was a key enabler for the implementation of web-based peer support interventions. The review by Lauckner and Hutchinson [12] and our review across similar chronic conditions suggests that the impact of face-to-face peer support interventions may be comparable with web-based peer support interventions, with web-based peer support interventions promoting accessibility and potentially reducing the stigma associated with face-to-face interventions.

### Limitations

We acknowledge some limitations of this scoping review. This review did not include primary mental health conditions and a variety of other disabilities. As previously mentioned, interventions that focused on mental illness were excluded from our list of chronic diseases, given that peer support interventions for this group may have unique features not generalizable to other patient populations with chronic disease, and a systematic review of digital peer support interventions for people with lived experience of a serious mental illness has recently been completed [86]. As web-based interventions also relate to computer science and information studies, there are additional databases that could have been included and would likely have identified a separate subfield of studies. Potential databases include IEEE and ACM, and they should be explored in future reviews on web-based interventions. This review did not look at the types of funding each study was provided with, and therefore, we cannot make definitive conclusions on whether interventions were scaled up. Furthermore, this review was limited to English language studies only and the published research literature. As a result, we likely have a bias toward studies from English-speaking countries, and we acknowledge that we likely excluded reports on other available, relevant programs (ie, but not published in peer-reviewed journals). Similarly, we excluded conference abstracts of posters or oral presentations (ie, without an accompanying, published article).

### Conclusions

The results of this review demonstrate that there is a limited, recent high-level evidence (ie, randomized controlled trials) on web-based peer support interventions. Where evidence exists, significant improvements in social participation, self-efficacy, and health-directed activity were demonstrated. However, these trials were limited to 4 conditions only: diabetes, HIV, cancer, and stroke. Thus, we recommend the study of web-based peer support in a much broader range of conditions. We further recommend the use of web-based peer support for older adults (ie, aged >70 years) with chronic conditions. We determined that some of the included studies incorporated a theoretical framework, and all forms of support—emotional, informational, and appraisal—were identified in the studies included in this review. Future peer support interventions should implement an underlying theory or model to inform interventions, which in turn would support the intended outcomes of the intervention. Future studies should also consistently report on the intervention characteristics, including the frequency and duration of the intervention, to promote replicability and to draw associations between intervention characteristics and specific outcomes. Overall, the results of this review have identified key gaps in the area of web-based peer support that will serve to inform the development, implementation, and evaluation of future programs.

## Appendix

Multimedia Appendix 1 Complete data set.

Multimedia Appendix 2 Frequency of delivery settings.

## Abbreviations

PHAC: Public Health Agency of Canada
PRISMA: Preferred Reporting Items for Systematic Reviews and Meta-Analyses
