# Supplementary figures and images for: Web-Based Peer Support Interventions for Adults Living With Chronic Conditions: Scoping Review
Source: JMIR Rehabil Assist Technol. 2021 May 25;8(2):e14321. doi: 10.2196/14321 (PMC8188320; doi:10.2196/14321)

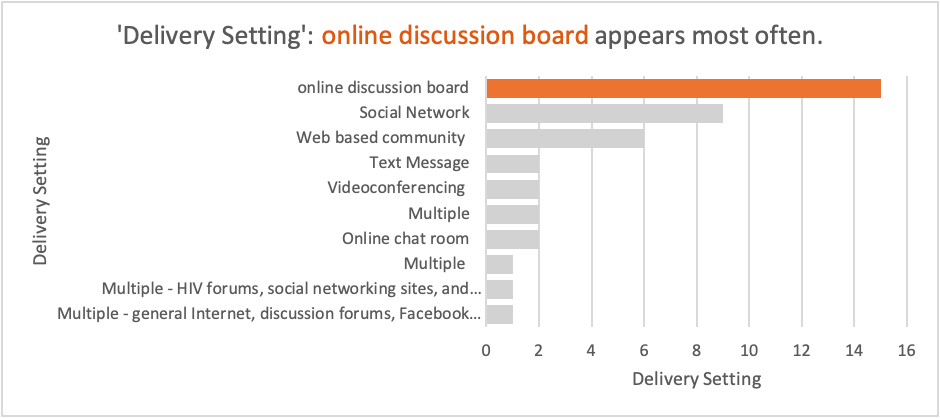

Supplement: Multimedia Appendix 2 [file rehab_v8i2e14321_app2.png]
